# Extracellular Vesicles from Vascular Endothelial Cells Promote Survival, Proliferation and Motility of Oligodendrocyte Precursor Cells

**DOI:** 10.1371/journal.pone.0159158

**Published:** 2016-07-12

**Authors:** Masashi Kurachi, Masahiko Mikuni, Yasuki Ishizaki

**Affiliations:** 1 Department of Molecular and Cellular Neurobiology, Gunma University Graduate School of Medicine, Maebashi, Japan; 2 Department of Psychiatry and Neuroscience, Gunma University Graduate School of Medicine, Maebashi, Japan; University of Torino, ITALY

## Abstract

We previously examined the effect of brain microvascular endothelial cell (MVEC) transplantation on rat white matter infarction, and found that MVEC transplantation promoted remyelination of demyelinated axons in the infarct region and reduced apoptotic death of oligodendrocyte precursor cells (OPCs). We also found that the conditioned medium (CM) from cultured MVECs inhibited apoptosis of cultured OPCs. In this study, we examined contribution of extracellular vesicles (EVs) contained in the CM to its inhibitory effect on OPC apoptosis. Removal of EVs from the CM by ultracentrifugation reduced its inhibitory effect on OPC apoptosis. To confirm whether EVs derived from MVECs are taken up by cultured OPCs, we labeled EVs with PKH67, a fluorescent dye, and added them to OPC cultures. Many vesicular structures labeled with PKH67 were found within OPCs immediately after their addition. Next we examined the effect of MVEC-derived EVs on OPC behaviors. After 2 days in culture with EVs, there was significantly less pyknotic and more BrdU-positive OPCs when compared to control. We also examined the effect of EVs on motility of OPCs. OPCs migrated longer in the presence of EVs when compared to control. To examine whether these effects on cultured OPCs are shared by EVs from endothelial cells, we prepared EVs from conditioned media of several types of endothelial cells, and tested their effects on cultured OPCs. EVs from all types of endothelial cells we examined reduced apoptosis of OPCs and promoted their motility. Identification of the molecules contained in EVs from endothelial cells may prove helpful for establishment of effective therapies for demyelinating diseases.

## Introduction

Demyelination as a result of white matter ischemia causes loss of brain functions [1,2], but there is no specific treatment so far. We previously showed that transplantation of brain microvascular endothelial cells (MVECs) greatly stimulated remyelination in the white matter infarct of the internal capsule (IC) induced by endothelin-1 (ET-1) injection and improved the behavioral outcome [3]. We also showed that MVEC transplantation reduced apoptotic death of oligodendrocyte precursor cells (OPCs) and that the conditioned medium (CM) from cultured MVECs (MVEC-CM) inhibited apoptosis of cultured OPCs [4]. These results indicate that MVECs produce and release some factors with a pro-survival activity on OPCs, and these factors might contribute to the recovery of the white matter infarct.

In the multicellular organism, survival, proliferation and differentiation of cells are controlled by the signals from other cells [5]. It is well known that various proteins such as growth factors, cytokines and adhesion molecules contribute to the cell-cell communication. Recently extracellular vesicles (EVs) secreted by cells have been reported to play an important role in the cell-cell communication [6–9]. EVs are released either through exocytosis of multivesicular bodies (MVBs) or through shedding of plasma membranes, and contain various proteins, lipids and nucleic acids such as mRNAs and microRNAs (miRNAs). These molecules are considered to play a role for the control of signal transduction and protein expression in the recipient cells.

In this study, we examined contribution of EVs contained in MVEC-CM to its inhibitory effect on apoptosis of cultured OPCs and obtained several lines of evidence for a role of EVs in the effect. We also found that these EVs promoted proliferation and motility of cultured OPCs. To determine whether these effects are shared by EVs from various endothelial cells, we examined the effect of EVs prepared from cultured rat brain microvascular (MVEC), human aortic (HAEC), dermal lymphatic microvascular (adult, HMVEC-dLyAd) and umbilical vein (HUVEC) endothelial cells on cultured OPCs. EVs isolated from these endothelial cells (ECs) reduced apoptotic cell death of cultured OPCs, and promoted their proliferation and motility. These results suggest a possibility that molecules contained in EVs from ECs contribute, to some extent at least, to the beneficial effect of MVEC transplantation on ischemic white matter infarct. Identification of the molecules may be useful for establishment of the therapeutic strategy against demyelinating diseases.

## Materials and Methods

### Animals

Sprague-Dawley (SD) rats (SLC, Japan) were used in this study (postnatal day 1–2 pups for OPC culture, and adults for MVEC cultures). All experiments were performed in accordance with the guidelines for Animal Experimentation at Gunma University Graduate School of Medicine and were approved by Gunma University Ethics Committee (Permit Number: 14–068). Rats were sacrificed by decapitation to obtain the brains.

### Cell culture

#### Primary culture of OPCs

Oligodendrocyte precursor cells (OPCs) were prepared from postnatal day 1–2 rat brain cortices by immunopanning as previously described [10]. Briefly, the meninges and superficial blood vessels were removed from cortices, and the cortical tissue was digested with 16.5 U/ml papain (Worthington) solution, triturated, and centrifuged. The pellet was suspended in the panning buffer (HBSS (Wako, Japan) containing 5 μg/ml insulin (Sigma) and 0.02% BSA (Sigma)). Dissociated cells were immunopanned on RAN-2 (ATCC), anti-galactocerebroside (IC-01, ECACC), and O4 [11] antibody-coated plates. OPCs isolated as O4-positive cells were suspended in serum-free medium: DMEM-F12 medium (Wako, Japan) containing 100 units/ml penicillin, 100 μg/ml streptomycin, 1 mM sodium pyruvate (all obtained from Gibco), 5 μg/ml insulin, 100 μg/ml apotransferrin, 100 μg/ml BSA, 62 ng/ml progesterone, 16 μg/ml putrescine, 40 ng/ml sodium selenite, and 30 μM N-acetyl cysteine (all obtained from Sigma). The cells were plated into poly-D-lysine (PDL) (Sigma)-coated tissue culture dish, supplemented with forskolin (Sigma) and CNTF (PeproTech). OPC proliferation was induced by the addition of NT-3 (PeproTech) and PDGF-AA (PeproTech) as mitogens. Cells were grown in 5% CO_2_ at 37°C. After several days, OPCs were harvested from dish using TrypLE Express (Gibco), washed, and suspended in the culture medium. OPCs were plated on PDL-coated 8-well slide glasses containing proliferation medium at the density of 12000 cells/cm^2^.

#### Primary culture of rat brain microvascular endothelial cells

Brain microvascular endothelial cells (MVECs) were prepared from adult rat cerebra [3,12]. In brief, the meninges and superficial blood vessels were removed, and the cortical tissue was digested with 0.15% dispase (Gibco) solution, triturated, and centrifuged. The pellet was digested again with 1 mg/ml of collagenase/dispase (Roche), washed, loaded onto continuous density gradient of 30% Percoll (GE Healthcare), and centrifuged. The fraction containing the microvessels was collected and centrifuged. The pellet was resuspended in the culture medium (EGM-2, Lonza) containing 4 μg/ml puromycin (Sigma) [13], and the cells were plated into collagen type I-coated dishes (Iwaki, Japan). Cultures were maintained in 5% CO_2_ at 37°C. After treatment with puromycin for 48 h, it was removed from the culture medium.

#### Human primary endothelial cells

Human umbilical vein endothelial cells (HUVEC) (Lonza, C-2517) and human aortic endothelial cells (HAEC) (Lonza, CC-2535) were cultured on collagen type I-coated dishes in endothelial cell growth medium (EGM-2, Lonza). Human dermal lymphatic microvascular endothelial cells–adult (HMVEC-dLyAd) (Lonza, CC-2810) were cultured on collagen type I-coated dishes in microvascular endothelial cell growth medium (EGM-2MV, Lonza).

#### Cell lines

Rat fibroblast-like cell line (Rat-1) and immortalized mouse cerebral endothelial cell line (bEnd.3) cells were cultured on collagen type I-coated dishes in DMEM (Wako, Japan) containing 10% fetal bovine serum (FBS).

### Analysis of the effect of MVEC-CM on cultured OPCs

To prepare MVEC-CM, MVECs (90–95% confluence) were washed with PBS, and then grown in serum-free medium for 24 h. CM was collected, and centrifuged to remove cell debris. The supernatant was filtered using 0.45μm filter before use in OPC experiments. To deplete EVs from MVEC-CM, MVEC-CM was ultracentrifuged at 130,000 × g at 4°C overnight, and the supernatant was collected as EV-depleted MVEC-CM (EV-dep-MVEC-CM). Neat CM was used in this experiment. OPCs plated on 8-well glass slides were wash with PBS, and then cultured in MVEC-CM or EV-dep-MVEC-CM for 3 days. As control, OPCs were cultured in serum-free medium.

### Isolation of EVs from culture media

For EV isolation, exosome-depleted FBS (System Biosciences) was used for cultures of ECs and Rat-1 cells to prevent contamination by EVs derived from FBS. After washing with PBS, cells were cultured in medium containing exosome-depleted FBS (MVECs, HAECs and HUVECs: EGM-2 containing 2% exosome-depleted FBS, HMVECs: EGM-2MV containing 5% exosome-depleted FBS, bEnd.3 and Rat-1: DMEM containing 10% exosome-depleted FBS) for 72 h. EV precipitation from conditioned media was performed using the exosome precipitation solution, ExoQuick-TC, (System Biosciences) according to the manufacturer’s protocol. Conditioned media were centrifuged at 3,000 × g for 15 minutes to remove cells and debris. The supernatant was mixed with ExoQuick solution, and refrigerated overnight. Samples were centrifuged at 1,500 × g for 30 minutes, and supernatant was removed. Samples were centrifuged again at 1,500 × g for 5 minutes, and all traces of supernatant were removed. EV pellet was resuspended in a small volume of buffer or serum-free medium depending on the following analyses.

### Analysis of the effect of EVs on cultured OPCs

Isolated EVs were suspended in serum-free medium at the protein concentrations of 3, 12.5, 50, and 200 μg/ml, and added to OPC cultures. OPCs plated on 8-well glass slides were wash with PBS, and then cultured in serum-free medium containing EVs for 2 days. As control, OPCs were cultured in serum-free medium.

### Cell survival assay

Cell death with the typical morphological features of apoptosis including pyknotic nuclei was assessed by staining cell nuclei with Hoechst 33342 (Sigma, 2.5 μM) for 40 min in 5% CO_2_ at 37°C. After incubation, samples were observed using a fluorescent microscope (Axioplan2, Zeiss) with Cooled CCD Camera (DP73, Olympus), and the number of pyknotic cells in each sample was counted.

### BrdU incorporation assay

To assess cell proliferation, bromodeoxyuridine (BrdU; 10 μM) (Roche Diagnostics) was added to the cultures for the last 4 h of culture, followed by fixation and staining with rat monoclonal BrdU antibody (abcam; 1:1000). Propidium iodide (Sigma, 2 μg/ml) was used for nuclear staining. Samples were observed using a fluorescent microscope (Axioplan2, Zeiss) with Cooled CCD Camera (DP73, Olympus), and the number of BrdU-positive cells in each sample was counted.

### Cell motility assay using reaggregated OPCs

OPC migration was measured using reaggregated OPCs [14]. OPC reaggregates were formed by plating OPCs on Petri dish (Corning) with shaking for 6 h. The reaggregated OPCs were then plated onto PDL-coated 8-well glass slides in MVEC-CM, EV-dep-MVEC-CM and serum-free medium containing EVs with 10 μM AraC. After incubation at 37°C in 5% CO_2_ for 16 h, the images of OPCs were captured using an inverted microscope (Primo-Vert, Zeiss). The distance of migration was calculated by measuring the change in size of the reaggregates over time, subtracting the average initial size of the reaggregates, and dividing the remaining distance in half. More than three aggregates in each condition were measured in three independent experiments.

### Protein assay and Western blot analysis

Protein concentration of solution including isolated EVs was measured using Protein Quantification Assay Kit (Takara Bio) according to the manufacturer’s protocol.

For Western blot analysis, EV pellet was lysed in a small volume of RIPA buffer (25 mM Tris-HCl, pH7.6, 150 mM NaCl, 1% NP-40, 1% sodium deoxycholate, 0.1% SDS). Samples were mixed with an equal volume of 2× SDS-PAGE sample buffer, heated at 95°C for 5 minutes, and separated by electrophoresis on 12.5% SDS-PAGE gels. Protein was transferred to a PVDF membrane. After washing with Tris-buffered saline containing 0.05% Tween-20 (TBS-T), membrane was blocked with 5% skim milk in TBS-T and incubated with primary antibodies diluted in 5% skim milk in TBS-T: a rabbit anti-CD63 antibody (System Biosciences; 1:500) at 4°C overnight, a mouse anti-rat CD9 antibody (BD Pharmingen; 1:250) at room temperature for 2 h, and a mouse anti-β-actin antibody (GenScript; 1:4000) at room temperature for 30 minutes. Membrane was washed, and incubated with horseradish peroxidase (HRP)-conjugated anti-rabbit or anti-mouse antibodies (GE Healthcare; 1:3000) diluted in TBS-T at room temperature for 60 minutes (30 minutes for actin). Signals were detected using ECL Prime Western blotting detection reagent (GE Healthcare) followed by image analysis using a CCD-based imager, FUSION-SL4-3500 (Vilber-Lourmat).

### Nanoparticle tracking analysis (NTA)

Isolated EVs were analyzed by nanoparticle tracking using NanoSight NS300 system (Malvern Instruments Ltd, Marvern, UK). EV samples were measured five times at 24 to 27°C, and particle sizes and concentrations in the samples were estimated.

### Statistical analysis

Statistical analysis was performed using R software (ver 3.2.2). Differences between groups were assessed with one-way analyses of variance followed by the *post hoc* Tukey–Kramer test. Student’s t-test was used to evaluate the differences between two groups. The error bars represent the standard errors. Cell counting was performed by persons unaware of the profile of samples.

## Results

### Depletion of EVs from MVEC-CM reduced its effect on OPC survival and motility

To examine whether EVs contained in MVEC-CM contribute to its inhibitory effect on apoptosis of cultured OPCs, OPCs were cultured in EV-depleted MVEC-CM (EV-dep-MVEC-CM).

**Fig 1:**
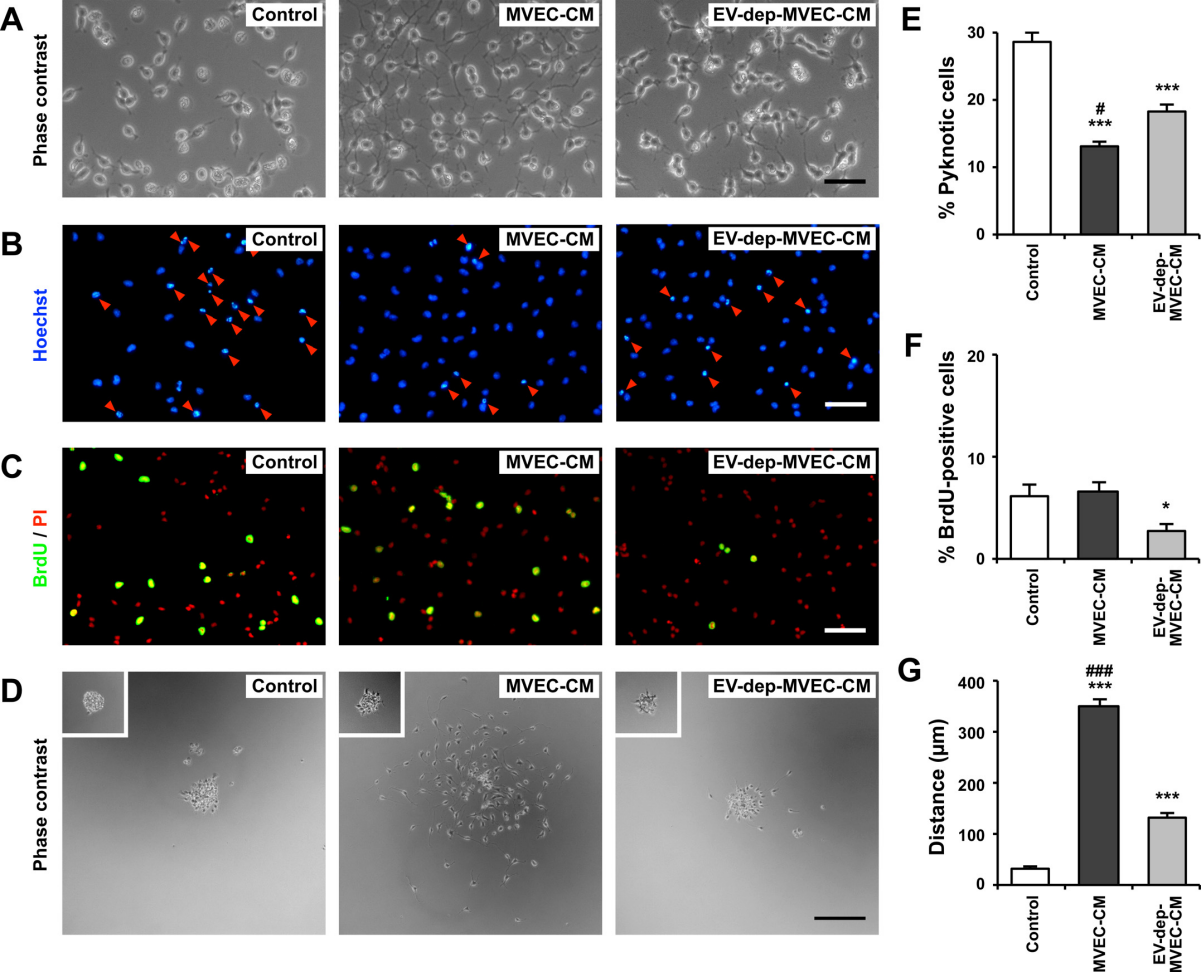
Removal of EVs from MVEC-CM reduced its effects on cultured OPCs. OPCs were cultured in MVEC-CM or EV-dep-MVEC-CM for 3 days (or overnight for cell motility assay). As control, OPCs were cultured in fresh serum-free medium. (A) Phase contrast images. Scale bar, 50 μm. (B) OPCs were stained with Hoechst 33342, and the nuclear morphology was observed. The number of pyknotic nuclei (red arrowheads) in MVEC-CM was smaller when compared to that of EV-dep-MVEC-CM. Scale bar, 50 μm. (C) After labeling with 10 μM BrdU for the last 4 h of culture, cells were fixed and stained with an anti-BrdU antibody. Cell nuclei were stained with propidium iodide (PI, red). Many BrdU-positive cells (green) were observed in MVEC-CM. Scale bar, 50 μm. (D) Phase contrast images of OPC migration 16 h after plating (*Insets*: OPC aggregates 1 h after plating). Scale bar, 200 μm. (E) All nuclei (pyknotic or non-pyknotic) were counted in a field of microscope and the fraction of pyknotic nuclei was determined, and results are shown as mean ± SE (N = 8 in each condition). Treatment with MVEC-CM significantly inhibited apoptotic cell death of OPCs. ***p<0.001 against control. #p<0.05 against EV-dep-MVEC-CM. (F) All cells were counted in a field of microscope and the fraction of BrdU-positive cells was determined, and results are shown as mean ± SE (N = 8 in each condition). The proportion of BrdU-positive cells in MVEC-CM was significantly larger when compared to EV-dep-MVEC-CM. *p<0.05 against control and MVEC-CM. (G) The distance of OPC migration in MVEC-CM was significantly larger when compared to EV-dep-MVEC-CM. Results are shown as mean ± SE (N = 4 in each condition). ***p<0.001 against control. ###p<0.001 against EV-dep-MVEC-CM. These experiments were repeated three times, and similar results were obtained each time. Typical experiments are shown here.

In MVEC-CM, many OPCs survived for 3 days with typical morphological features of healthy cells (Fig 1A). In contrast, in serum-free medium (control) and EV-dep-MVEC-CM, many OPCs died with the typical morphological features of apoptosis including pyknotic nuclei. As we reported previously, MVEC-CM had no apparent effect on OPC differentiation [4].

To analyze cell survival, OPCs were stained with Hoechst 33342, a cell-permeant nuclear staining dye, and the nuclear morphology was observed. The proportion of pyknotic (apoptotic) nuclei (red arrowheads) in MVEC-CM and EV-dep-MVEC-CM were both smaller when compared to control (Fig 1B and 1E). The proportion of pyknotic nuclei in EV-dep-MVEC-CM, however, were significantly larger when compared to MVEC-CM (Fig 1E).

We then examined the effect of MVEC-CM on OPC proliferation. The proportion of BrdU-positive cells in MVEC-CM was the same as that in control, but that in EV-dep-MVEC-CM was significantly smaller when compared to MVEC-CM and control (Fig 1C and 1F).

We also examined the effect of MVEC-CM on OPC motility. MVEC-CM greatly promoted motility of OPCs when compared to control and EV-dep-MVEC-CM, though EV-dep-MVEC-CM still showed a small but significant promoting effect on OPC motility (Fig 1D and 1G).

Taken together, MVEC-CM promoted OPC survival and motility, and removal of EVs from MVEC-CM by ultracentrifugation significantly reduced its effect on OPC survival and motility.

### EVs from MVEC-CM were taken up by cultured OPCs and promoted their survival, proliferation, and motility

**Fig 2:**
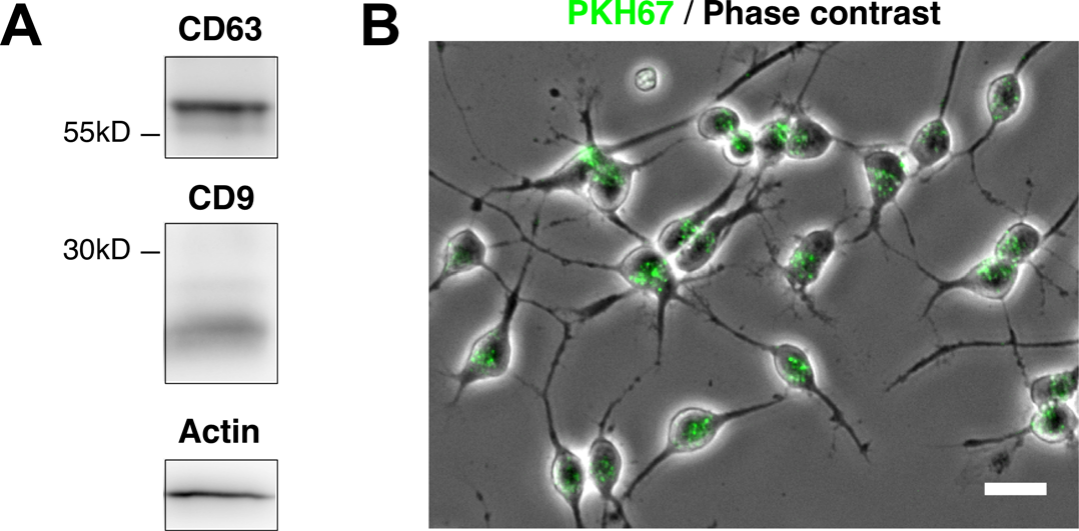
Cultured OPCs incorporated isolated MVEC-EVs. Presence of exosomal markers (CD63 and CD9), and actin, a cytoskeletal protein, in isolated MVEC-EVs was confirmed by Western blot (A). MVEC-EVs were labeled with fluorescent dye PKH67, and added to OPC culture. PKH67-labeled EVs were immediately taken up by OPCs, and many small vesicles (green) were found in OPCs (B). Scale bar: 20 μm.

As the above experiments suggested a possibility that MVEC-derived EVs influence the behavior of OPCs, we isolated EVs from MVEC-CM to examine directly whether EVs affect cultured OPCs. When EVs prepared from MVEC-CM (MVEC-EVs) were analyzed by Western immunoblotting for CD63 and CD9, transpanin family proteins, they were positive for these exosome markers (Fig 2A).

When MVEC-EVs were labeled with PKH67, a fluorescent dye, and added to OPC cultures, PKH67-labeled EVs were immediately taken up by OPCs, and many small vesicles were found within OPCs (Fig 2B). This result supports the possibility that MVEC-EVs affect cultured OPCs.

Nanoparticle tracking analysis (NTA) revealed the modes of 95.2 ± 4.7 nm, 92.1 ± 2.5 nm, and 105.0 ± 4.5 nm in particle distribution of MVEC-EVs, HUVEC-EVs, and Rat-1-EVs respectively, which, on the whole, fall within the size of exosomes. NTA also revealed the ratio of particle number to protein concentration of EV samples; 2.48 × 10^10^ particles/μg protein, 3.03 × 10^10^ particles/μg protein, 2.42 × 10^10^ particles/μg protein for MVEC-EVs, HUVEC-EVs, and Rat-1-EVs respectively.

**Fig 3:**
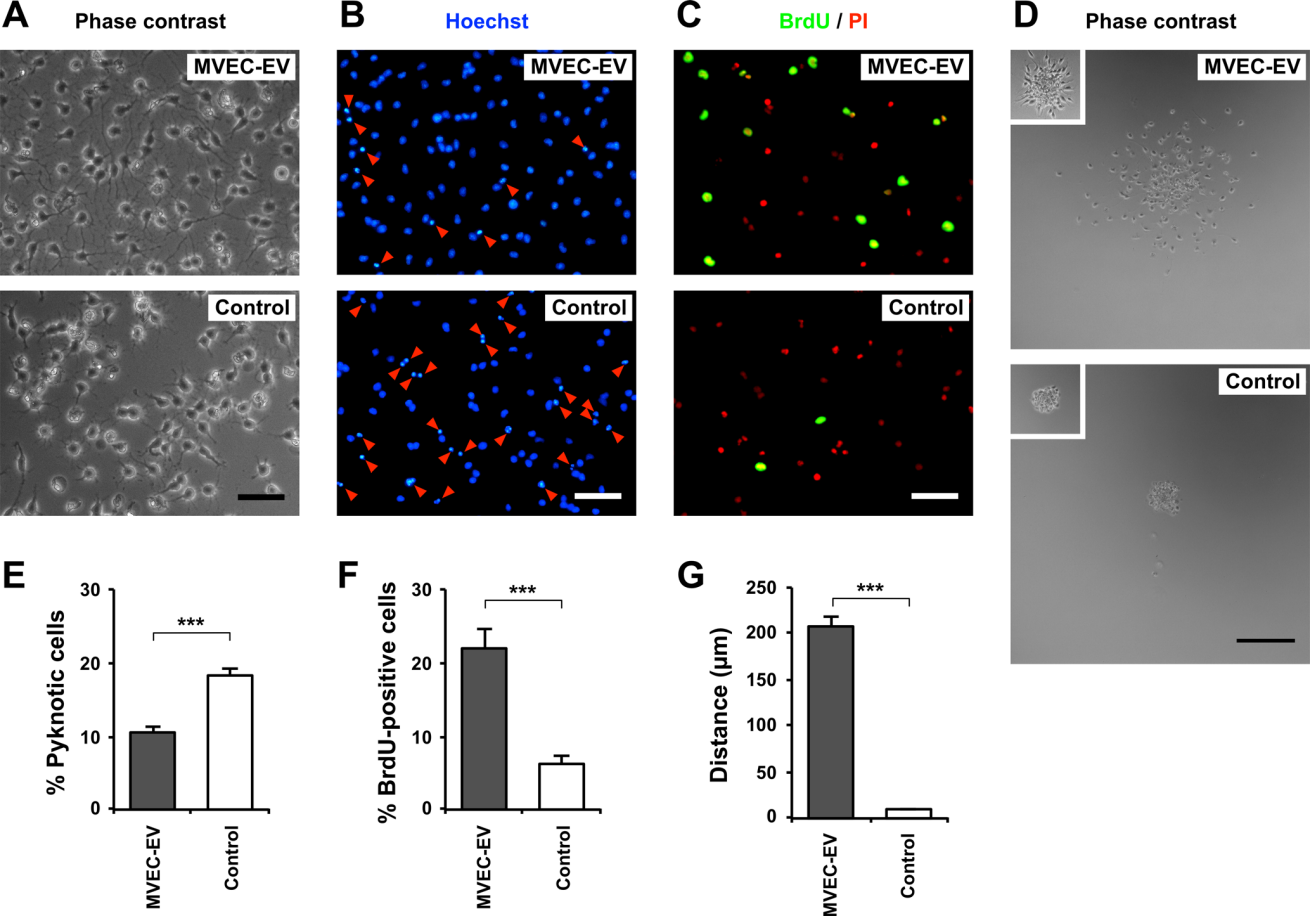
MVEC-EVs promoted OPC survival, proliferation and motility. EVs isolated from MVEC-CM were suspended in fresh serum-free medium at 50 μg/ml of proteins and added to OPC culture, which were maintained for 2 days (or overnight for cell motility assay). As control, OPCs were cultured in fresh serum-free medium without EVs. (A) Phase contrast images. Scale bar, 50 μm. (B) OPCs were stained with Hoechst 33342, and the nuclear morphology was observed. The proportion of pyknotic nuclei (red arrowheads) in OPCs with MVEC-EVs was smaller when compared to control. Scale bar, 50 μm. (C) After labeling with BrdU for the last 4 h of culture, cells were fixed and stained with an anti-BrdU antibody. Cell nuclei were stained with propidium iodide (PI, red). Many BrdU-positive cells (green) were observed in the presence of MVEC-EVs. Scale bar, 50 μm. (D) Phase contrast images of OPC migration 16 h after plating (*Insets*: OPC aggregates at 1 h after plating). Scale bar, 200 μm. (E) The proportion of pyknotic nuclei in the presence of MVEC-EVs was significantly smaller when compared to control. Results are shown as mean ± SE (N = 8 in each condition). ***p<0.001. (F) The proportion of BrdU-positive cells in the presence of MVEC-EVs was significantly larger when compared to control. Results are shown as mean ± SE (N = 8 in each condition). ***p<0.001. (G) The distance of OPC migration in the presence of MVEC-EVs was significantly larger when compared to control. Results are shown as mean ± SE (N = 4 in each condition). ***p<0.001. These experiments were repeated three times, and similar results were obtained each time. Typical experiments are shown here.

We then examined the effect of MVEC-EVs on cultured OPCs. After 2 days in culture in the presence of EVs (50 μg/ml protein), there was a significant difference in the morphology of OPCs. In the presence of MVEC-EVs, OPCs looked much healthier with sturdy processes when compared to control (Fig 3A). To estimate cell death, OPCs were stained with Hoechst 33342 dye. There were significantly less pyknotic nuclei in the presence of MVEC-EVs when compared to control (Fig 3B and 3E), suggesting that MVEC-EVs inhibit apoptosis of OPCs. To determine the proportion of proliferating cells, BrdU incorporation assay was used. More BrdU-positive OPCs were seen in the presence of MVEC-EVs when compared to control (Fig 3C and 3F), suggesting MVEC-EVs promote OPC proliferation. We also examined the effect of MVEC-EVs on OPC motility and found that MVEC-EVs greatly promoted the motility (Fig 3D and 3G). Taken together, MVEC-EVs promoted OPC survival, proliferation, and motility.

**Fig 4:**
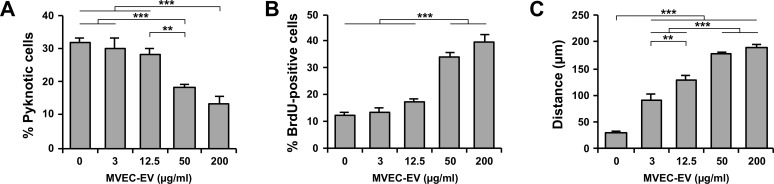
MVEC-EVs promoted OPC survival, proliferation, and motility in a dose-dependent manner. EVs isolated from MVEC-CM were suspended in fresh serum-free medium at 0, 3, 12.5, 50, and 200 μg/ml proteins and added to OPC culture, which were maintained for 2 days (or overnight for cell motility assay). (A) The proportion of pyknotic nuclei decreased at doses ≥ 50 μg/ml. Results are shown as mean ± SE (N = 8 in each condition). **p<0.01 and ***p<0.001. (B) The proportion of BrdU-positive cells increased at doses ≥ 50 μg/ml. Results are shown as mean ± SE (N = 8 in each condition).***p<0.001. (C) The distance of OPC migration gradually increased in a dose-dependent manner. Results are shown as mean ± SE (N = 4 in each condition). **p<0.01 and ***p<0.001. These experiments were repeated three times, and similar results were obtained each time. Typical experiments are shown here.

Next, we examined the dose-dependent effects of MVEC-EVs on OPC behavior. Isolated MVEC-EVs were suspended in serum-free medium and add to OPC culture at 0, 3, 12.5, 50, and 200 μg/ml proteins. After 2 days in culture, the proportion of pyknotic nuclei was reduced at doses ≥ 50 μg/ml (Fig 4A). The proportion of BrdU-positive OPCs at doses ≥ 50 μg/ml increased as compared with lower doses (0, 3, 12.5 μg/ml) (Fig 4B). For OPC motility assay, reaggregated OPCs were cultured overnight at 0, 3, 12.5, 50, and 200 μg/ml proteins. MVEC-EVs increased the distance of OPC migration in a dose-dependent manner (Fig 4C). These results clearly revealed that MVEC-EVs promoted OPC survival, proliferation and motility in a dose-dependent manner.

### EVs derived from endothelial cells promoted OPC survival, proliferation and motility

We then examined whether these effects on cultured OPCs are shared by EVs from endothelial cells. EVs were prepared from various types of endothelial cells (bEnd.3: immortalized mouse cerebral endothelial cell line, HAEC: human aortic endothelial cells, HMVEC (-dLyAd): human dermal lymphatic microvascular endothelial cells–adult, HUVEC: human umbilical vein endothelial cells). EVs were also prepared from rat fibroblast-like cell line (Rat-1). EVs prepared from CM of cultures of each cell type were suspended in serum-free medium and added to OPC culture at 50 μg/ml of EV proteins, and maintained for 2 days. As control, OPCs were cultured in serum-free medium without EVs.

**Fig 5:**
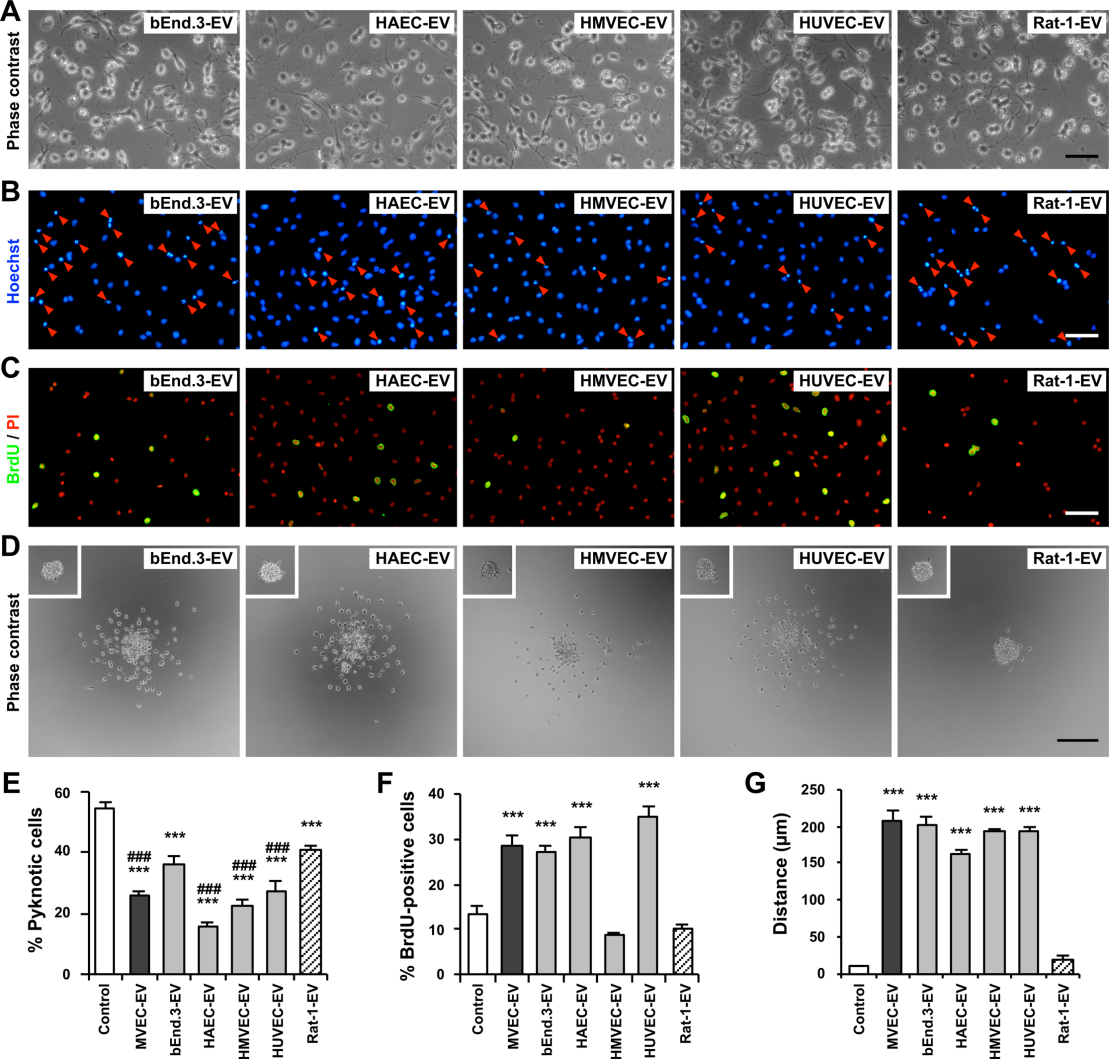
EC-derived EVs promoted OPC survival, proliferation and migration. EVs were isolated from conditioned media of various types of endothelial cells (ECs) or Rat-1 fibroblastic cells, suspended in fresh serum-free medium and added to OPC cultures (50 μg/ml of proteins), which were maintained for 2 days. As control, OPCs were cultured in fresh serum-free medium without EVs. (A) Phase contrast images. Scale bar, 50 μm. (B) OPCs were stained with Hoechst 33342, and the nuclear morphology was observed. The proportion of pyknotic nuclei (red arrowheads) in OPCs with EC-derived EVs was smaller when compared to Rat-1-EVs. Scale bar, 50 μm. (C) After labeling with BrdU for the last 4 h of culture, cells were fixed and stained with an anti-BrdU antibody. Cell nuclei were stained with propidium iodide (PI, red). Many BrdU-positive cells (green) were observed in HUVEC-EV. Scale bar, 50 μm. (D) Phase contrast images of OPC migration 16 h after plating (*Insets*: OPC aggregates 1 h after plating). Scale bar, 200 μm. (E) The proportion of pyknotic nuclei in the presence of EC-derived EVs (except for bEnd.3-EVs) was significantly smaller when compared to those in the presence of Rat-1-EVs. Results are shown as mean ± SE (N = 8 in each condition). ***p<0.001 against control. ###p<0.001 against Rat-1-EVs. (F) The proportion of BrdU-positive cells in the presence of EC-derived EVs (except for HMVEC-EVs) was significantly larger when compared to Rat-1-EVs. Results are shown as mean ± SE (N = 8 in each condition). ***p<0.001 against control and Rat-1-EVs. (G) EVs derived from ECs significantly promoted OPC migration, when compared to control and Rat-1-EVs. Results are shown as mean ± SE (N = 4 in each condition). ***p<0.001 against control and Rat-1-EVs.

After 2 days in culture, in the presence of HAEC- and HUVEC-EVs, the shape of OPCs were similar to that in MVEC-EVs (Fig 3A), having sturdy processes, as shown in Fig 5A. In the presence of MVEC-EVs, HAEC-EVs, HMVEC-EVs and HUVEC-EVs, pyknotic nuclei were fewer when compared to bEnd.3-EVs and Rat-1-EVs, although the latter two EVs also significantly reduced pyknotic nuclei when compared to control (Fig 5B and 5E). Treatment with EC-derived EVs, except for bEnd.3-EVs, significantly inhibited apoptotic cell death of OPCs when compared to Rat-1-EVs. In the presence of EC-derived EVs, except for HMVEC-EVs, the proportion of BrdU-positive cells was significantly larger when compared to control (Fig 5C and 5F). There was no significant difference in the number of BrdU-positive cells in the presence of HMVEC-EVs or Rat-1-EVs when compared to control (Fig 5F). Treatment of reaggregated OPCs with EC-derived EVs promoted their motility, and OPCs spread radially from initial reaggregates, while Rat-1-EVs had no effect on OPC motility (Fig 5D and 5G).

## Discussion

We confirmed our previous study reporting that MVEC-CM reduced apoptotic cell death of OPCs [4], and found that MVEC-CM promoted their motility (Fig 1). ECs are active and dynamic cells producing various biologically active molecules in response to various stimuli. It is well known that FGF-2, PDGF and VEGF are among important soluble proteins playing roles in OPC survival, proliferation and differentiation [15–21], and are released from ECs [21–26]. In addition, adhesion proteins such as laminin and fibronectin also affect OPC behavior [27,28], and are secreted by ECs [29]. MVEC-CM possibly contains these molecules, and these molecules in soluble form may exert various effects on cultured OPCs. In this study, we examined the effect of EVs prepared from rat brain MVECs on the behavior of rat OPCs, and obtained several lines of evidence supporting a role for MVEC-derived EVs in the cell–cell communication between MVECs and OPCs. First, inhibitory effect of MVEC-CM on OPC apoptosis were significantly reduced by removal of MVEC-EVs by ultracentrifugation. Second, fluorescently labeled MVEC-EVs are readily taken up by cultured OPCs. And third, MVEC-EVs promoted survival, proliferation and motility of cultured OPCs.

MVEC-CM showed no enhancement of OPC proliferation, while MVEC-EVs promoted their proliferation. There are two possibilities for this discrepancy. One is that we used neat CM for our experiments and the concentrations of EVs contained in CM were too low to exert their effect. The other is that some soluble factors inhibitory for OPC proliferation are contained in CM and these negate the promoting effect of EVs. Although the finding that EV-dep-MVEC-CM reduced OPC proliferation when compared to control supports the latter possibility, further studies are necessary to test these possibilities.

Recently there are several reports on EVs in the central nervous system (CNS). It has recently been reviewed that neuronal EVs play roles in neuronal-glial communication and contribute to neuronal development and disease mechanisms [30,31]. The *in silico* interactomics analyses, which were based on molecular profile of EVs derived from human brain microvascular endothelial cells (HBECs), showed that HBEC-EVs may engage in numerous cell-surface interactions with astrocytes and neurons [32]. Thus there is a possibility that similar EV-mediated communication might occur between ECs and OL-lineage cells.

EVs released from ECs are reported to contain various miRNAs [33,34]. It has already been reported that some miRNAs are closely related to the development of OL-lineage cells [35–38]. MicroRNA (miR-17-92) cluster is important in cell cycle, proliferation, apoptosis and other pivotal processes [39]. It is known that this microRNA cluster is highly expressed in ECs [40], and that, in OPCs, this cluster promotes cell survival and proliferation by influencing Akt signaling [41]. Therefore, there is a possibility that members of miR-17-92 cluster contained in MVEC-EVs promote survival and proliferation of OPCs. MicroRNA-221/222 (miR-221/222) are also highly expressed in ECs, and one of their target genes is the cyclin-dependent kinase inhibitor, p57/Kip2 (Cdkn1c) [42]. In OL-lineage cells, p57/Kip2 plays a crucial role in cell proliferation by inhibiting CyclinE-cdk2 complex formation. Reducing p57/Kip2 in OPCs, therefore, promotes their proliferation [43]. So there is another possibility that miR-221/222 contained in MVEC-EVs reduces expression of p57/Kip2 in OPCs and thereby promotes OPC proliferation. Mass spectrometric analysis revealed that various types of proteins are present in EVs released from ECs [32,33]. Proteomics analyses showed that cell surface proteins, including adhesion molecules, extracellular matrix (ECM) molecules and other cell-cell interacting molecules, were present in EVs [32,44]. It is well known that ECM molecules regulate various aspects of cellular behavior [45], and it was reported that ECM molecules affected the growth properties of OPCs [28]. Thus the effect of MVEC-CM on OPCs observed in this study may be mediated by ECM molecules present in MVEC-EVs.

Several cell types, derived from different species and organs, were used in this study. EVs prepared from all cell types we examined reduced apoptosis of cultured OPCs (Fig 5). EVs secreted by primary cultured ECs (MVEC, HAEC, HMVEC, HUVEC) were more effective on OPC survival than those from immortalized ECs (bEnd.3) or fibroblast-like cells (Rat-1). This result suggests the possibility that EVs prepared from each cell type contain the same molecules with a pro-survival activity on OPCs. Differences in effects of EVs on OPC survival may reflect the quantity of the molecules present in EVs, and EVs secreted by primary cultured ECs may contain them in larger quantity. Regarding OPC proliferation, HMVEC-EVs and Rat-1-EVs showed no effect. Rat-1-EVs didn’t affect OPC motility either. These results suggest that several different molecules (proteins and/or RNAs) in the EVs were independently or synergistically affect OPC behavior (survival, proliferation and motility). Further studies are necessary to identify the molecules responsible for the effects of MVEC-EVs on OPCs.

Recently therapeutic potentials of EVs for CNS diseases have been suggested [46,47]. Systemic treatment of stroke with EVs derived from multipotent mesenchymal stromal cells (MSCs) promoted neurovascular plasticity and functional recovery after stroke in rats [48]. miR-219, microRNA contained in EVs derived from dendritic cells and serum, stimulated remyelination in rats [49,50]. Our study demonstrates that EVs prepared from ECs promote survival, proliferation, and motility of OPCs. Although it remains undetermined whether EVs released from transplanted MVECs are involved in its beneficial effect on white matter infarct, identification of molecules present in EVs from ECs may prove useful in establishment of effective therapeutic strategy against demyelinating diseases. In this respect, our finding that HUVEC-EVs affect OPC behaviors is promising, as it is relatively easy to prepare EVs from HUVECs in quantity necessary for proteomic analysis and RNA profiling.
